# Isosorbide Nitrate-Assisted Microcatheter Navigation for Successful Embolization of a Foramen Magnum Dural Arteriovenous Fistula

**DOI:** 10.7759/cureus.87548

**Published:** 2025-07-08

**Authors:** Shoichiro Tsuji, Kotaro Tatebayashi, Shinya Minamimoto, Yoji Kuramoto, Shinichi Yoshimura

**Affiliations:** 1 Neurological Surgery, Hyogo Medical University, Nishinomiya, JPN; 2 Neurological Surgery, Aoyama Neurosurgical Hospital, Fujiidera, JPN

**Keywords:** ascending pharyngeal artery, foramen magnum dural arteriovenous fistula, isosorbide dinitrate, pharmacologic vasodilation, transarterial embolization

## Abstract

This case report describes the use of pharmacologic vasodilation with intra-arterial isosorbide dinitrate to facilitate endovascular treatment of a foramen magnum dural arteriovenous fistula in a man in his 40s. The fistula was supplied by the posterior meningeal artery arising from the right vertebral artery and the hypoglossal branch of the ascending pharyngeal artery (APA). Catheterization of the APA was challenging due to its tortuous course and small caliber. To address this, 2 mg of isosorbide dinitrate was administered intra-arterially, resulting in effective vasodilation and allowing smooth microcatheter navigation. Embolization was then successfully performed using 12.5% n-butyl-2-cyanoacrylate, leading to a significant reduction in shunt flow. Follow-up angiography at three months confirmed complete obliteration of the fistula, and the patient remained neurologically intact, with no perioperative or delayed complications. This case highlights the potential utility of intra-arterial vasodilators, such as isosorbide dinitrate, in overcoming anatomical challenges during transarterial embolization and suggests their broader applicability in complex neuroendovascular procedures.

## Introduction

We report what we believe to be the first documented case of using intra-arterial isosorbide dinitrate to facilitate microcatheter navigation through the ascending pharyngeal artery (APA) in the treatment of a foramen magnum dural arteriovenous fistula (FM-dAVF). FM-dAVF is a rare vascular lesion located at the craniocervical junction that can present with life-threatening complications such as subarachnoid hemorrhage, progressive myelopathy, or cranial nerve dysfunction [1]. These lesions are typically supplied by meningeal branches arising from regional arteries, including the vertebral and occipital arteries, with the APA frequently playing a critical role [2].

However, the APA presents significant anatomical challenges for endovascular treatment due to its small caliber, tortuous course, and close proximity to cranial nerves IX through XII. Navigating a microcatheter through the hypoglossal branch of the APA often requires distal access beyond the hypoglossal canal to safely and effectively reach the dural feeders. Yet, the vessel’s narrow diameter and angulation may prevent adequate catheter advancement, limiting embolic penetration and increasing the risk of incomplete occlusion or recurrence [3].

While the posterior meningeal artery (PMA) may occasionally provide an alternative route to the target lesion and may offer relatively easier navigation compared to the APA, it is often absent or diminutive, limiting its utility in many cases. Thus, achieving safe and reliable distal microcatheter positioning within the APA remains a key procedural challenge in the treatment of FM-dAVF.

In this context, we describe a novel strategy to address these anatomical limitations: the intra-arterial administration of isosorbide dinitrate to induce targeted pharmacologic vasodilation. This technique enabled smooth and deep advancement of the microcatheter through the hypoglossal branch of the APA, ultimately allowing for effective transarterial embolization (TAE). To our knowledge, this represents one of the first reported uses of intra-arterial isosorbide dinitrate in this setting. Our findings suggest that pharmacologic vasodilation may serve as a valuable adjunct in neuroendovascular procedures involving tortuous or high-risk arterial anatomy.

## Case presentation

**Figure 1:**
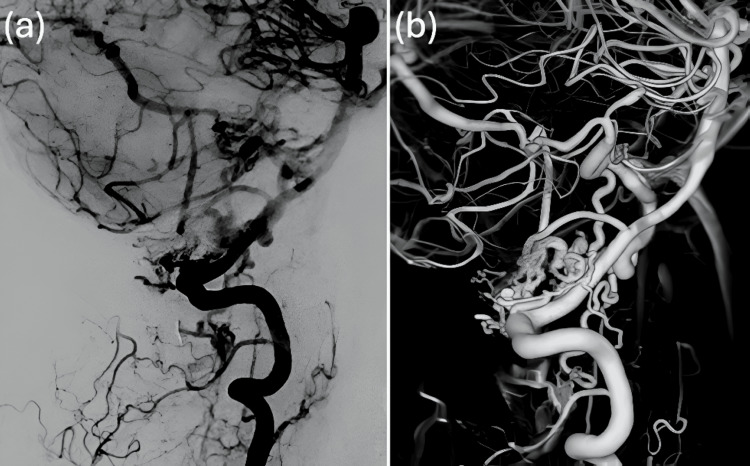
Right VAG (a) and 3D angiography (b) showing the right PMA as a feeder of the FM-dAVF FM-dAVF, foramen magnum dural arteriovenous fistula; PMA, posterior meningeal artery; VAG, vertebral artery angiography

**Figure 2:**
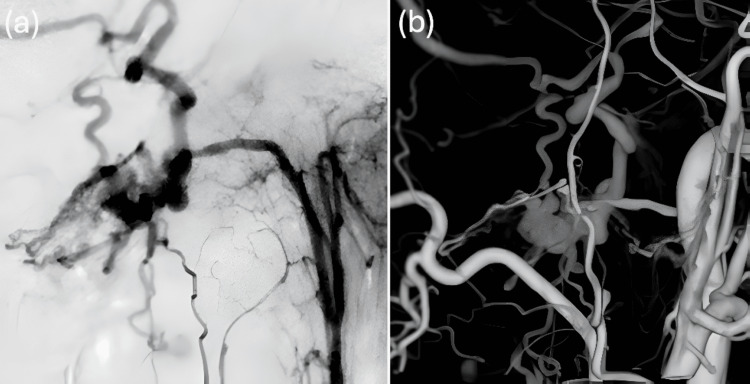
Selective lateral view of right APA angiography (a) and 3D right CCAG (b) demonstrating the APA as an additional contributor to the dAVF APA, ascending pharyngeal artery; CCAG, common carotid artery angiography; dAVF, dural arteriovenous fistula

**Figure 3:**
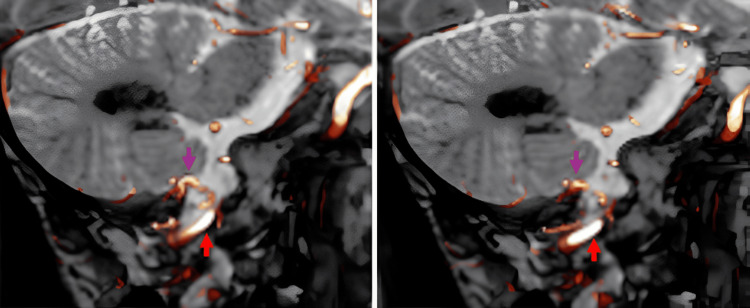
Sagittal fused images of CISS MRI, right VAG, and CCAG revealing a focal caliber change (purple arrow) at the suspected shunt point CCAG, common carotid artery angiography; CISS MRI, constructive interference in steady state MRI; VAG, vertebral artery angiography

A man in his 40s presented with posterior neck pain that had been present for three weeks. The pain was non-radiating and not associated with trauma or systemic symptoms. Head MRI revealed abnormal vasculature at the craniocervical junction, prompting referral to our institution for further evaluation. Cerebral angiography identified an FM-dAVF supplied by the posterior meningeal branch of the right vertebral artery (VA) (Figure 1a, 1b) and the ipsilateral APA (Figure 2a, 2b). The shunt point was fed by both the right VA and the right APA, with caliber changes noted along the involved segments (Figure 3). Venous drainage occurred via the vein of the great horizontal fissure and the petrosal vein into the transverse sinus.

Although common symptoms of FM-dAVF include progressive myelopathy, subarachnoid hemorrhage, and lower cranial nerve palsies, none were observed in this case. The patient was neurologically intact at presentation; however, treatment was recommended due to the potential risk of progression to a more aggressive form.

After a thorough discussion of therapeutic options, including direct surgical interruption of the draining vein and endovascular embolization, the patient opted for endovascular treatment. To allow seamless conversion to surgical management in the event of endovascular failure, the procedure was planned in a hybrid operating room.

**Figure 4:**
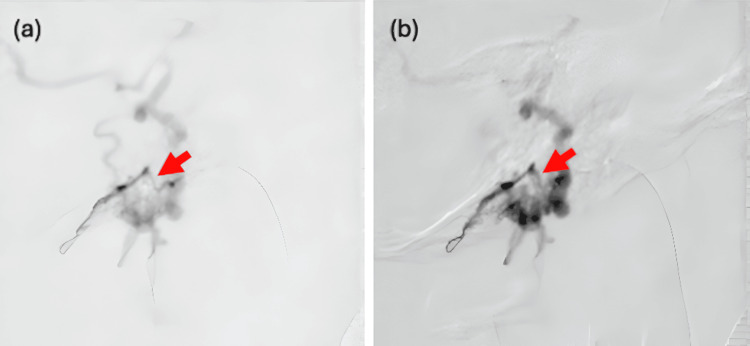
DSA images before and after intra-arterial administration of isosorbide dinitrate demonstrating vasodilation of the APA (a) The DeFrictor Nano microcatheter was unable to advance beyond the hypoglossal canal due to severe tortuosity (red arrow: constricted segment). (b) Following intra-arterial isosorbide dinitrate administration, the vessel was visibly dilated, allowing successful microcatheter navigation (red arrow: dilated segment). APA, ascending pharyngeal artery; DSA, digital subtraction angiography

TAE under general anesthesia was selected. The goal was to navigate a microcatheter beyond the hypoglossal canal to reach the shunt point. A 5-Fr guide catheter was introduced via the right femoral artery and advanced to the right CCA. A Guidepost catheter (Tokai Medical Products, Kasugai, Japan) was then directed into the right APA. However, initial attempts to advance various microcatheters to the target site were unsuccessful due to the vessel’s severe tortuosity and small caliber (Figure 4a).

To overcome this challenge, a DeFrictor Nano microcatheter (Medico’s Hirata, Osaka, Japan) was used in combination with intra-arterial pharmacologic vasodilation. A 2 mL aliquot of isosorbide dinitrate (5 mg/10 mL) was diluted tenfold and infused at a rate of 1 mL/s, delivering a total dose of 2 mg (Figure 4b).

**Figure 5:**
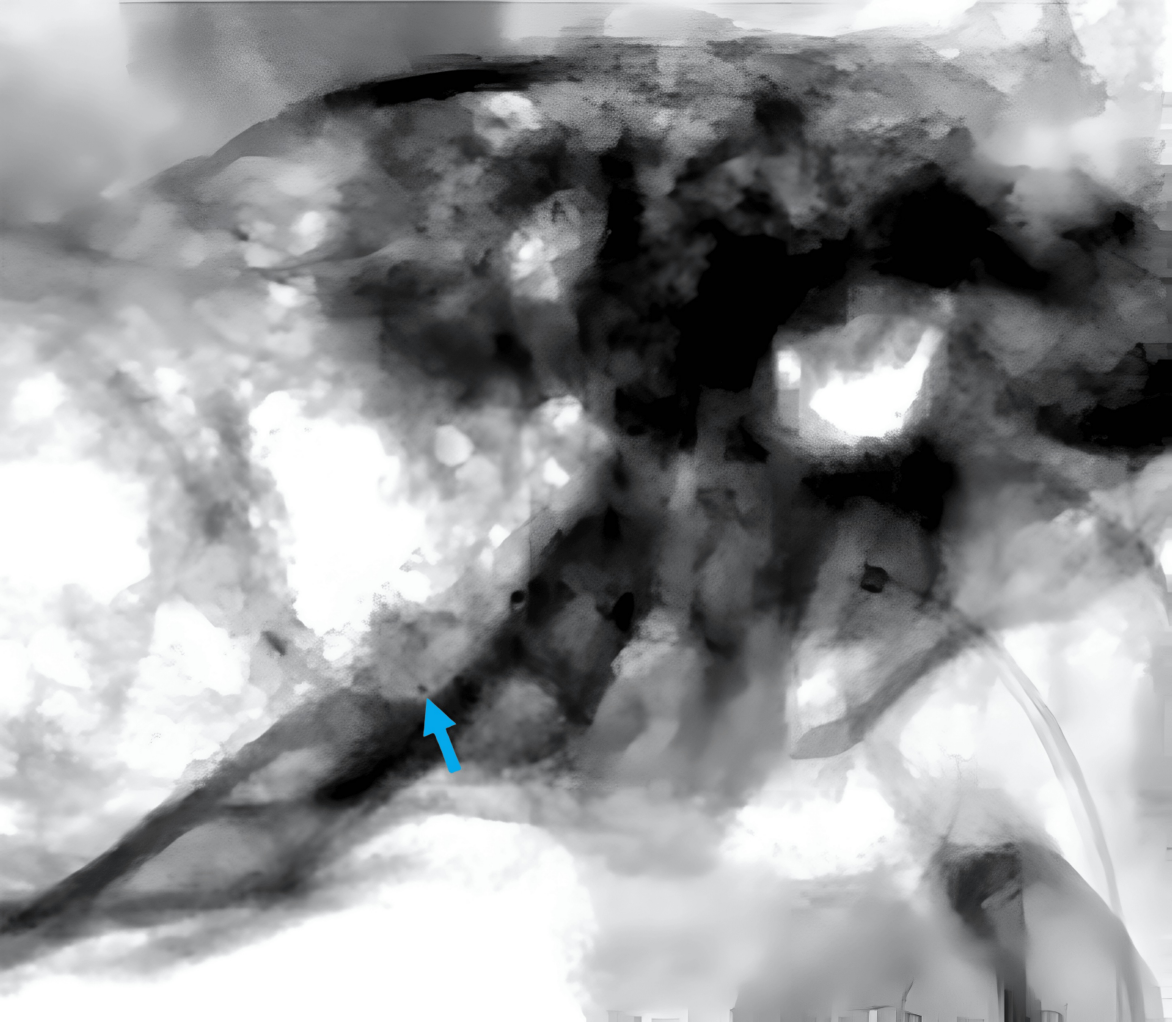
Successful distal advancement of the microcatheter beyond the hypoglossal canal The blue arrow indicates the distal marker of the microcatheter, confirming its position past the hypoglossal canal.

This strategy successfully facilitated microcatheter navigation beyond the hypoglossal canal to reach the shunt site (Figure 5).

**Figure 6:**
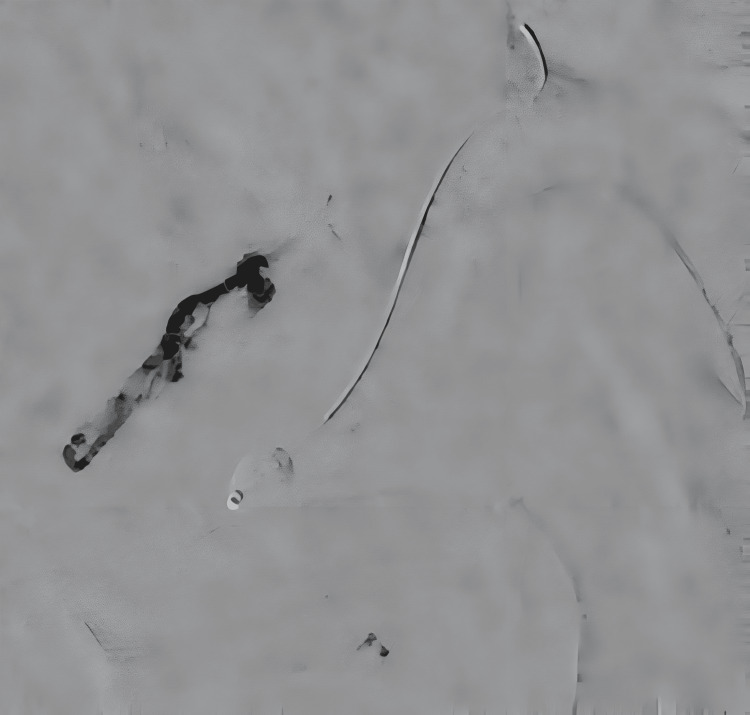
NBCA embolization under flow control using a SHORYU 2 HR balloon catheter The balloon was inflated to achieve flow arrest, allowing safe and effective injection of NBCA for embolization. NBCA, n-butyl-2-cyanoacrylate

For flow control, a 4-Fr guide catheter was introduced via the right radial artery to access the right VA. A SHORYU 2 HR 4/7 balloon catheter (Kaneka Medical Products, Tokyo, Japan) was positioned at the bifurcation of the dural branch of the VA. After balloon inflation, 0.12 cc of 12.5% n-butyl-2-cyanoacrylate was injected through the microcatheter into the hypoglossal branch of the APA (Figure 6). Although angiography demonstrated a marked reduction in shunt flow, complete occlusion was not achieved. Due to concerns about the risk of over-embolization, the procedure was terminated at that point.

Intra-arterial administration of isosorbide dinitrate (2 mg) resulted in angiographically apparent vasodilation within approximately one minute, which lasted an estimated five to seven minutes. Throughout the embolization, selective lateral angiography of the right APA was continuously performed for monitoring. The total procedure duration was approximately 173 minutes, with a fluoroscopy time of 42.4 minutes and a total contrast volume of 273 mL. Hemodynamic and neurophysiological parameters were continuously monitored during the procedure. No bradycardia, hypotension, or new neurological symptoms were observed following intra-arterial isosorbide dinitrate administration.

**Figure 7:**
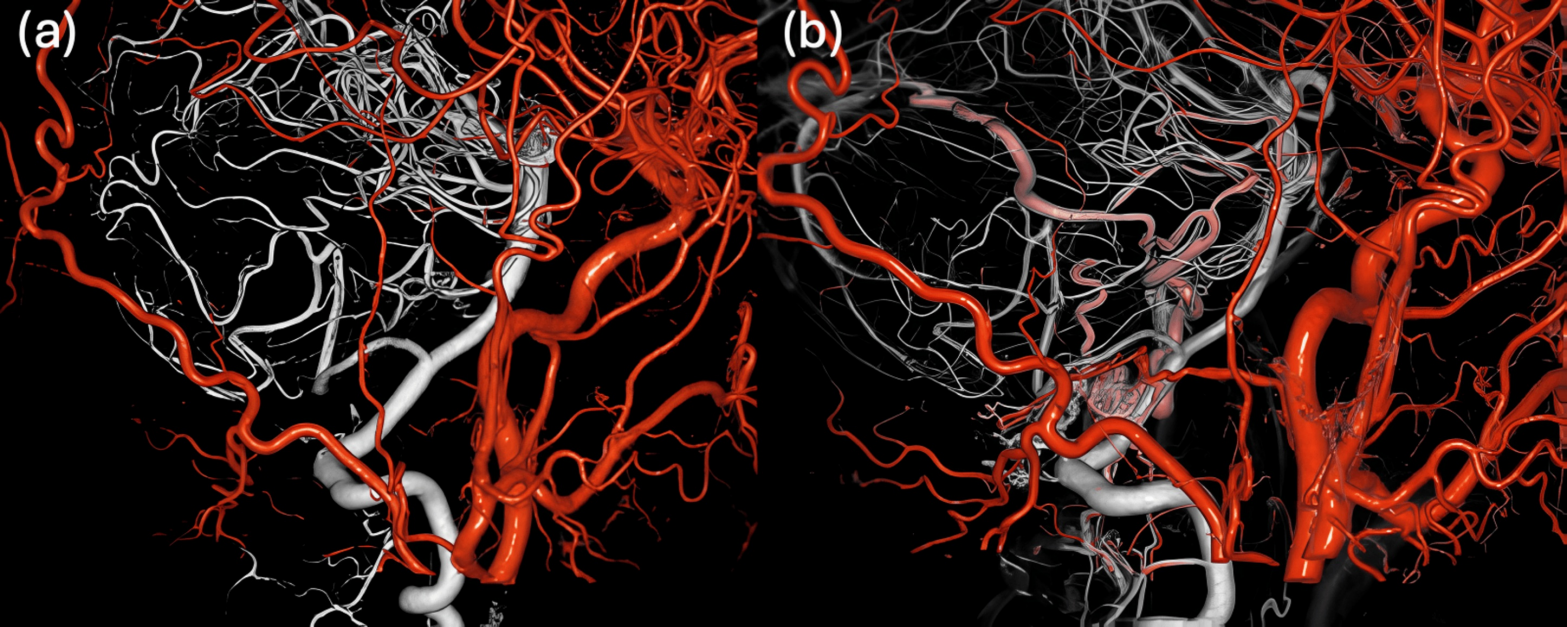
Fusion 3D angiography combining CCAG and VAG (a) Follow-up fusion 3D angiography at three months post-treatment shows complete resolution of the arteriovenous shunt, with no evidence of recurrence or residual arteriovenous flow. (b) Initial fusion 3D angiography prior to treatment, demonstrating the presence of the arteriovenous shunt. CCAG, common carotid artery angiography; VAG, vertebral artery angiography

The patient was discharged home on postoperative day seven with a Modified Rankin Scale score of 0 and no neurological deficits. Digital subtraction angiography at the three-month follow-up confirmed complete resolution of the shunt, with no evidence of recurrence (Figure 7a), in contrast to the pre-treatment findings (Figure 7b).

## Discussion

This case report highlights two key clinical insights: first, the anatomical challenges associated with navigating the APA during embolization of an FM-dAVF; and second, the potential role of intra-arterial isosorbide dinitrate as a pharmacologic adjunct to improve microcatheter navigability in such procedures.

Dural arteriovenous fistulas (dAVFs) are abnormal connections between arteries and veins within the dura mater. When located near the foramen magnum - the base of the skull - these lesions can result in serious neurological complications due to impaired venous drainage. The APA frequently supplies this region, but accessing it is often difficult due to its small diameter and deep, tortuous course.

The optimal treatment approach for FM-dAVFs remains debated. While microsurgical disconnection has long been considered definitive [4], advances in endovascular techniques have enabled safer and more effective treatment, especially when selective embolization is achieved through distal arterial feeders such as the APA [5]. Although embolization of craniocervical junction dAVFs carries a reported complication rate of approximately 15.8% [6], the structural similarities between FM-dAVFs and spinal dAVFs support the growing consensus that endovascular treatment can offer both efficacy and safety [7].

In the present case, the APA was selected as the access route over the PMA due to safety concerns. The PMA is known to have potentially hazardous anastomoses with the VA, increasing the risk of embolic reflux into the VA or even the basilar artery (BA). Catastrophic complications, including BA occlusion, have been reported following Onyx reflux during PMA embolization [8]. While the APA is less commonly used for transarterial access due to its narrow caliber, complex anatomy, and risk of cranial nerve palsy, it can still serve as a viable and safe alternative when approached with careful technique and appropriate safeguards [3].

Navigating a microcatheter through the APA, particularly into the neuromeningeal trunk and beyond the hypoglossal canal, is often technically demanding. Balloon-assisted Onyx embolization via the APA has been reported as effective but challenging, requiring deep catheter positioning to avoid reflux into non-target branches [9]. In this case, initial attempts to advance the catheter through the APA were unsuccessful due to the vessel’s tortuosity and small diameter.

To address this challenge, intra-arterial isosorbide dinitrate, a nitrate-based vasodilator commonly used to prevent radial artery spasm, was administered. While its use in neurovascular interventions has primarily been described in the management of cerebral vasospasm [10], to our knowledge, no prior reports have detailed its application in improving microcatheter navigation during TAE of dAVFs. Isosorbide dinitrate has a rapid onset and short half-life (approximately one to four minutes), with vasodilatory effects typically lasting five to 10 minutes [11]. Reported doses include 0.5 mg for radial artery spasm [11] and up to 3 mg for internal carotid artery vasospasm [10]. In this case, a 2 mg intra-arterial dose enabled successful distal catheter advancement beyond the hypoglossal canal.

Pharmacologic vasodilation with isosorbide dinitrate likely facilitated smoother catheter manipulation by temporarily increasing vessel diameter and reducing resistance along the catheter track. This not only improved procedural efficiency but also minimized the risk of vessel injury and vasospasm - critical concerns when navigating narrow, high-risk arterial territories such as the APA [12]. As demonstrated in this case, intra-arterial isosorbide dinitrate may be a valuable adjunct to enhance microcatheter access and procedural safety in complex neuroendovascular interventions.

Beyond FM-dAVFs, intra-arterial vasodilators such as isosorbide dinitrate may have broader utility in neuroendovascular procedures. For example, in the treatment of arteriovenous malformations, accessing distal, tortuous feeders can be technically challenging, and transient vasodilation may aid catheter navigation. Similarly, vasospasm, whether spontaneous or iatrogenic, can hinder device advancement and may benefit from targeted pharmacologic relaxation. Additionally, in transradial neurointerventions, isosorbide dinitrate is already routinely used to prevent radial artery spasm; its use could potentially be extended to facilitate access in other small-caliber or tortuous vessels encountered during cerebral angiography or embolization. These potential applications merit further investigation.

As shown in Table 1, the current literature provides limited data on intra-arterial nitrate use in neurointervention. A prospective registry or pilot study could help define optimal dosing protocols, evaluate safety profiles, and assess clinical outcomes across a range of neurovascular conditions [13-17].

**Table 1:** Reported cases of dAVFs treated via the APA approach This table summarizes previously reported cases of dAVFs managed through the APA, highlighting clinical presentation, arterial feeders, and embolic agents used. The APA was frequently utilized either alone or in combination with other feeders, such as the VA or OA. Various embolic materials, including NBCA, Onyx, and PVA, were employed, with most cases reporting favorable clinical outcomes. APA, ascending pharyngeal artery; dAVF, dural arteriovenous fistula; IBCA, isobutyl cyanoacrylate; NBCA, n-butyl-2-cyanoacrylate; OA, occipital artery; PVA, polyvinyl alcohol; VA, vertebral artery

Reference	Sex/age	Presentation	Feeder	Treatment
[13]	Male/50	Myelopathy	VA, APA	Embolization (PVA, IBCA)
[14]	Male/57	Myelopathy	APA	Embolization (NBCA)
	Male/50	Tinnitus, CN VI palsy	APA, OA	Embolization (PVA)
[15]	Male/53	Myelopathy	APA	Embolization (glue)
[16]	Male/50	Myelopathy	APA	Embolization (PVA)
[17]	Male/55	Myelopathy	APA, VA	Embolization (Onyx)
	Female/49	Myelopathy	OA, VA, APA	Embolization (Onyx)

Compared with other vasodilators like verapamil and nicardipine, which are calcium channel blockers with longer onset and duration of action, isosorbide dinitrate offers a more rapid onset and shorter half-life, providing the advantage of controlled, transient vasodilation (Table 2) [18-20].

**Table 2:** Pharmacologic properties of intra-arterial vasodilators This table compares key pharmacologic characteristics of commonly used intra-arterial vasodilators in neurointervention, including isosorbide dinitrate, verapamil, and nicardipine. Parameters include onset time, duration of effect, typical intra-arterial dose ranges, and relative risk of neurotoxicity.

Agent	Class	Onset (approx.)	Duration	Typical intra-arterial dose	Neurotoxicity risk	Reference
Isosorbide dinitrate	Nitrate	<1 minute	5-10 minutes	0.5-2 mg	Low	[18]
Verapamil	Calcium channel blocker	2-5 minutes	30-60 minutes	5-20 mg	Moderate	[19]
Nicardipine	Calcium channel blocker	1-3 minutes	15-45 minutes	1-5 mg	Low-moderate	[20]

Further investigation into the comparative efficacy, hemodynamic effects, and procedural applications of these agents would be valuable for guiding neuroendovascular practice.

In summary, this case illustrates the importance of strategic route selection in FM-dAVF embolization and presents the first reported use of intra-arterial isosorbide dinitrate to facilitate microcatheter advancement through the APA. Further studies are warranted to establish the efficacy, safety, and standardized dosing of pharmacologic vasodilation in the treatment of cranial dAVFs and other complex neurovascular lesions.

## Conclusions

This case demonstrates the utility of intra-arterial isosorbide dinitrate as a pharmacologic adjunct to facilitate microcatheter navigation through the APA during embolization of an FM-dAVF. The strategic choice of the APA over the more hazardous PMA, combined with targeted vasodilation, allowed for the safe and effective delivery of the embolic agent to the distal shunt site. This approach minimized the risk of embolic reflux and improved microcatheter stability in a challenging anatomical setting.

To our knowledge, this represents the first documented use of isosorbide dinitrate to enhance distal microcatheter access during TAE of a cranial dAVF. The success of this technique suggests that pharmacologic vasodilation may serve as a valuable adjunct in neuroendovascular procedures where vessel tortuosity or small caliber limits catheter advancement. Furthermore, this case highlights the importance of tailoring access strategies to individual vascular anatomy and utilizing pharmacologic tools to broaden the feasibility and safety of embolization in high-risk areas. Further clinical studies are needed to validate the safety, determine optimal dosing, and assess the broader applicability of this technique in the management of complex dAVFs.
